# Acute Effects of Short-Term Warm Water Immersion on Arterial Stiffness and Central Hemodynamics

**DOI:** 10.3389/fphys.2021.620201

**Published:** 2021-02-04

**Authors:** Jun Sugawara, Tsubasa Tomoto

**Affiliations:** ^1^Human Informatics and Interaction Research Institute, National Institute of Advanced Industrial Science and Technology, Tsukuba, Japan; ^2^Institute for Exercise and Environmental Medicine, Texas Health Presbyterian Hospital Dallas, Dallas, TX, United States; ^3^Department of Neurology and Neurotherapeutics, University of Texas Southwestern Medical Center, Dallas, TX, United States

**Keywords:** warm water immersion, arterial stiffness, aortic hemodynamics, pulse wave analysis, femoral arterial blood flow

## Abstract

Warm water immersion (WWI) has a potentially favorable effect on vascular health. However, the effects of short-term WWI on vascular function and central hemodynamics remain unclear. The present study aimed to determine the acute effects of short-term WWI on arterial stiffness and central hemodynamics in healthy men. Ten healthy men (27–57 years, 44 ± 12 years of mean age) underwent 5-min WWI (40–41°C) at the heart level. Systemic hemodynamics and tympanic temperature were monitored during WWI. Furthermore, pulse wave velocity (PWV) and aortic hemodynamics were measured before and 10 min after WWI. Cardiac output (CO) (via the Modelflow method) increased (*P* = 0.037), whereas tympanic temperature did not change (*P* = 0.879) during WWI. After 5-min WWI, heart rate (HR) and brachial diastolic blood pressure (BP) were significantly decreased. Aortic and leg PWV were decreased by 7.5 and 3.1%, respectively (*P* = 0.006 and *P* = 0.040). Femoral arterial blood flow was increased by 45.9% (*P* = 0.002), and leg vascular resistance was decreased by 29.1% (*P* < 0.001). Regarding central hemodynamic variables (estimated by general transfer function), aortic BP and augmentation index (AIx) did not change significantly, but the subendocardial viability ratio (SEVR), an index of coronary perfusion, was increased (*P* = 0.049). Our results indicate that a short-term WWI acutely improves aortic and peripheral arterial stiffness and coronary perfusion. Further studies to determine the interaction between the residual effect of a single bout of short-term WWI and chronic change (e.g., adaptation) in arterial stiffness and central hemodynamics are needed.

## Introduction

Cardiovascular disease (CVD) is a leading cause of death in the world (Luscher, 2016). Currently, effective strategies for preventing CVD have been developed extensively (Luscher, 2016). CVD is characterized by vascular dysfunction, including impaired endothelial-dependent vasodilation and arterial stiffening (Benjamin et al., 2017). In this line, regular physical activity (i.e., aerobic exercise) is one of the most established treatments of the primary prevention of CVD because it may restore impaired vascular function (Arnett et al., 2019). However, not all populations can engage in physical activities. Thus, substitute effects of physical activity on vascular hemodynamics are needed for individuals who have physical disabilities.

Along this line, passive heat therapy may have the potential of the similar effects of exercise on cardiovascular health. Water immersion *per se* evokes cardiovascular reflexes (Bonde-Petersen et al., 1992). The earlier study reported that under the thermoneutral condition, the increased central blood volume induces the increase in stroke volume (SV) and the concomitant decrease in heart rate (HR) by 15%; eventually, cardiac output (CO) raised 18%. On the other hand, under the hot water condition (43.8°C), HR increased by 32%, and hence CO increases by 44%, suggesting the temperature effect overriding the immersion effect. Since thermal stimulation elicits vasodilatory substances, such as nitric oxide (Taylor and Bishop, 1993; Minson et al., 2001), repetitive accelerated systemic circulation may potentiate long-term improvements in vascular function (i.e., endothelial function, arterial stiffness). Indeed, Brunt et al. (2016) have reported that repetitive warm water immersion (WWI) at the heart level (40.5°C for 1 h, four to five times per week, 8 weeks) improved endothelium-dependent dilatation and central arterial stiffness. However, effective approaches of WWI (i.e., duration) on vascular health are not fully established.

The acute effects of WWI on pulse wave velocity (PWV), an index of arterial stiffness, are inconsistent in previous studies. For example, Kosaki et al. (2015) demonstrated that heating of the lower leg via the foot bath decreased leg PWV but not aortic PWV. On the other hand, the beneficial impact of heat stimulation which accompanies core temperature elevation on central arterial stiffness is often not observed. Using a water-perfused tube-lined suit, Ganio et al. (2011) evaluated central and peripheral arterial stiffness during whole-body heating by 49°C water through the suit. This intervention evoked 0.5, 1.0, and 1.5°C elevation in core temperature from pre-heat-stress baseline for 36 ± 5, 60 ± 11, and 77 ± 15 min of exposure, respectively, but they did not induce significant changes either in aortic or leg PWV. Likewise, Schlader et al. (2019) found no significant change in central arterial stiffness in older adults during whole-body heating by 49°C water and 1.3°C of core temperature elevation (approximately 18 min). Passive heat stress is accompanied by sympathetic activation, dehydration, and elevation of blood viscosity that may mask arterial destiffening (Low et al., 2011; Gagnon et al., 2015; Stojadinovic et al., 2015).

A recent prospective survey of the emergency events related to bathing reveals that consciousness disturbance and exhaustion are the most frequent symptoms and which are presumably attributed to hyperthermia and dehydration (Suzuki et al., 2019). From the standpoint of safety, to determine the influence of short term rather than prolonged bathing using warm water on vascular function should be encouraged. Accordingly, the present study aimed to determine the acute effects of short-term WWI on arterial stiffness and central hemodynamics in healthy men. We hypothesized that short-term WWI decreases aortic and peripheral arterial stiffness and alters central hemodynamics in healthy men.

## Materials and Methods

### Subjects

Ten healthy men (age of 44 ± 12 years [27–57 years], the height of 172.0 ± 4.2 cm, the weight of 64.6 ± 8.7 kg, body mass index of 21.8 ± 2.7 kg/m^2^, body fat of 15.4 ± 6.8%, mean ± standard deviation [SD]) were studied. Exclusion criteria were medicated individuals, having overt CVDs as assessed by medical history, and current smokers. The sample size was based on that of similar previous studies (Ganio et al., 2011; Kosaki et al., 2015) and confirmed statistical power. Among the limited number of subjects, to determine relationships between PWV and variables of interest effectively, we recruited individuals who had various arterial stiffness. All experimental procedures and protocols conformed to the Declaration of Helsinki and were approved by the institutional review board (#2016-683, National Institute of Advanced Industrial Science and Technology). All subjects provided written informed consent before participation.

### Experimental Protocol

Subjects abstained from alcohol intake and strenuous physical activity for at least 24 h before the study and food and caffeine intake 3 h before the study. All measurements were performed in a temperature-controlled single room (24–26°C). Upon arrival, subjects underwent height and weight assessments, which were followed by supine rest for more than 10 min and the main experimental protocol which consisted of 5-min WWI and two (pre- and post-WWI) vascular measurements as listed in the Supplementary Table 1. Each subject moved between the bed and the bathtub (approximately 3 m apart) on foot. During WWI, each subject who wore only swim shorts (no shirt) sat on the floor of the bathtub. The water level was set at the manubrium of sternum of each individual. Water temperature was monitored and maintained automatically at approximately 40–41°C during the 5-min bathing. The water temperature was decided based on the previous study which reported the favorable chronic effect of passive heat therapy on endothelial function and arterial stiffness (Brunt et al., 2016). The tympanic membrane temperature was measured before WWI and 30 s before the ending of WWI by the infrared tympanic thermometer (Genius 2, GOVIDIEN, Mansfield, MA, United States). Wiping out his body quickly after WWI, subjects walked to the bed and took a 10-min quiet supine rest, which was followed by the post-WWI vascular measurement.

### Vascular Measurements

#### Systemic Hemodynamics and Pulse Wave Velocity

Arterial pressure waveform was continuously recorded at the left middle finger by Finometer system (Finometer MIDI, Finapres Medical Systems, Amsterdam, Netherlands) during WWI and stored on a computer using a data acquisition system (PowerLab, AD Instrument) at the 1000 Hz of sampling rate. Throughout the WWI, subject was asked to keep the left hand at the heart level: on the side-table out of the bathtub. SV, CO, and total peripheral resistance (TPR) were estimated with a non-invasive blood pressure (BP) measurement device incorporated Modelflow-based hemodynamics measurement software (Finometer Model-2, Finapres Medical Systems, Amsterdam, Netherlands). The validity of the Modelflow method to derive hemodynamic measurements has been established in a variety of conditions (Wesseling et al., 1993; Sugawara et al., 2003). HR, brachial BP, and PWV were measured with a vascular testing device equipped with an electrocardiogram, phonocardiogram, oscillometric extremities cuffs (form PWV/ABI; Colin Medical Technology, Komaki, Japan), and an applanation tonometry sensor unit (TU-100; Colin Medical Technology, Komaki, Japan), as previously described (Kosaki et al., 2015; Sugawara et al., 2016, 2019). Carotid and femoral arterial pressure waveforms were simultaneously recorded by two applanation tonometry sensors incorporating an array of 15 micro-piezoresistive transducers. Briefly, PWV (=arterial path length/pulse transit time) was obtained between the carotid and femoral regions (e.g., aorta) and between the femoral and ankle regions (e.g., leg) as an indirect index of local arterial stiffness. Arterial path lengths were assessed with a straight distance measurement over the surface of the body using a steel measure. The carotid-femoral body surface straight distance multiplied 0.8 was applied for aortic PWV calculation (Van Bortel et al., 2012).

#### Central Hemodynamic Variables

**FIGURE 1:**
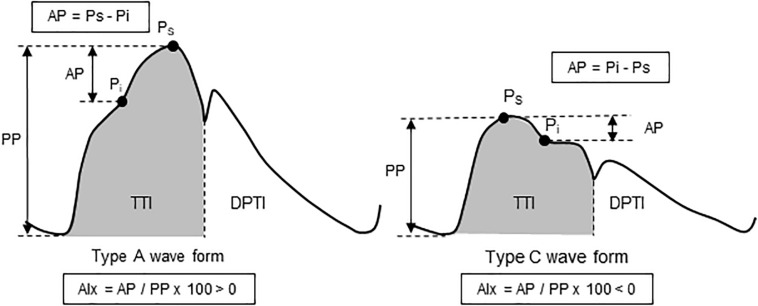
Schema of aortic pulse wave analysis. Augmentation pressure (AP) is the additional pressure added to the forward wave by the reflected wave. Augmentation index (AIx) is defined as the AP as a percentage of pulse pressure. The dicrotic notch (DN) represents the closure of the aortic valve and is used to calculate ejection duration. Aortic tension-time index (TTI) and diastolic pressure-time index (DPTI) reflect the myocardial oxygen demand and the blood supply to the heart, respectively. These are calculated as the areas under the curve of aortic pressure waveform in systole and diastole, respectively. The subendocardial viability ratio (SEVR) is the ratio of DPTI to TTI.

Aortic pressure waveforms were synthesized from the carotid pressure waveforms with the generalized transfer function using SphygmoCor system (Model MM3, AtCor Medical, West Ryde, NSW, Australia). Measurements of aortic pulse wave analysis are presented in Figure 1. Carotid and aortic BP were calibrated with the brachial mean arterial pressure and diastolic BP (Armentano et al., 1995). Aortic augmentation pressure (AP) was defined as the difference between the first systolic peak (or shoulder) and the second systolic peak pressures (Kelly et al., 1989). Aortic augmentation index (AIx) was calculated as the percent ratio of aortic AP to aortic pulse pressure and standardized for an HR of 75 bpm. From the synthesized aortic pulse, areas under the systolic and diastolic parts of the curves [the time-tension index (TTI) and diastolic pressure-time index (DPTI)] were obtained. Subendocardial viability ratio (SEVR = DPTI/TTI^∗^100) was calculated as the ratio of the myocardial oxygen demand and the blood supply to the heart (Buckberg et al., 1972; Tanaka et al., 2016).

#### Leg Hemodynamics

Femoral artery blood flow was measured with an ultrasound machine equipped with a high-resolution multi-frequency linear-array transducer (14 MHz) (CX50xMATRIX, Philips Ultrasound, Bothell, WA, United States), as we previously reported (Sugawara et al., 2007). Briefly, the longitudinal two-dimensional and Doppler ultrasound images were consecutively obtained below the inguinal ligament, 2–3 cm above the bifurcation into the profundus and superficial branches. For mean blood flow velocity measurements, a probe insonation angle was calibrated to 60°. The sample volume was adjusted to cover the width of the common femoral artery to encompass the entire lumen of the vessel, and the cursor was set at mid-vessel. Five-second B-mode movies and 5-s Doppler images were stored at least two of each as a DICOM file for later offline analysis. To eliminate the inter-investigator variability, the same person analyzed ultrasound images with computerized image-analysis software (ImageJ, NIH) in a blind manner. The arterial diameter was determined by a perpendicular measurement from the media/adventitia interface of the near-wall to the lumen/intima interface of the far wall of the vessel. A mean diameter was calculated based on the relative time periods of the systolic (1/3) and diastolic (2/3) BP phases and was used to represent the cross-sectional area. Three measurements of arterial lumen diameter were taken per frame and averaged. Blood flow was calculated as (mean blood velocity) × (circular area) × 6 × 10^4^ (with the constant 6 × 10^4^ being the conversion factor from m/s to L/min). Data reported are the time averages of ≥10 measurements for all variables. Femoral arterial shear stress (SS) was calculated using the following equation: 8 × blood viscosity (assumed to be 0.035 dyn × s/cm^2^) × mean blood velocity/baseline diameter (at end-diastole) (Dhindsa et al., 2008).

### Statistical Analyses

A paired *t*-test was applied for comparison between pre- and post-WWI. A simple correlation analysis was performed to determine the relations of interests. All data are reported as mean ± SD. Statistical significance was set *a priori* at *P*-values less than 0.05.

## Results

**FIGURE 2:**
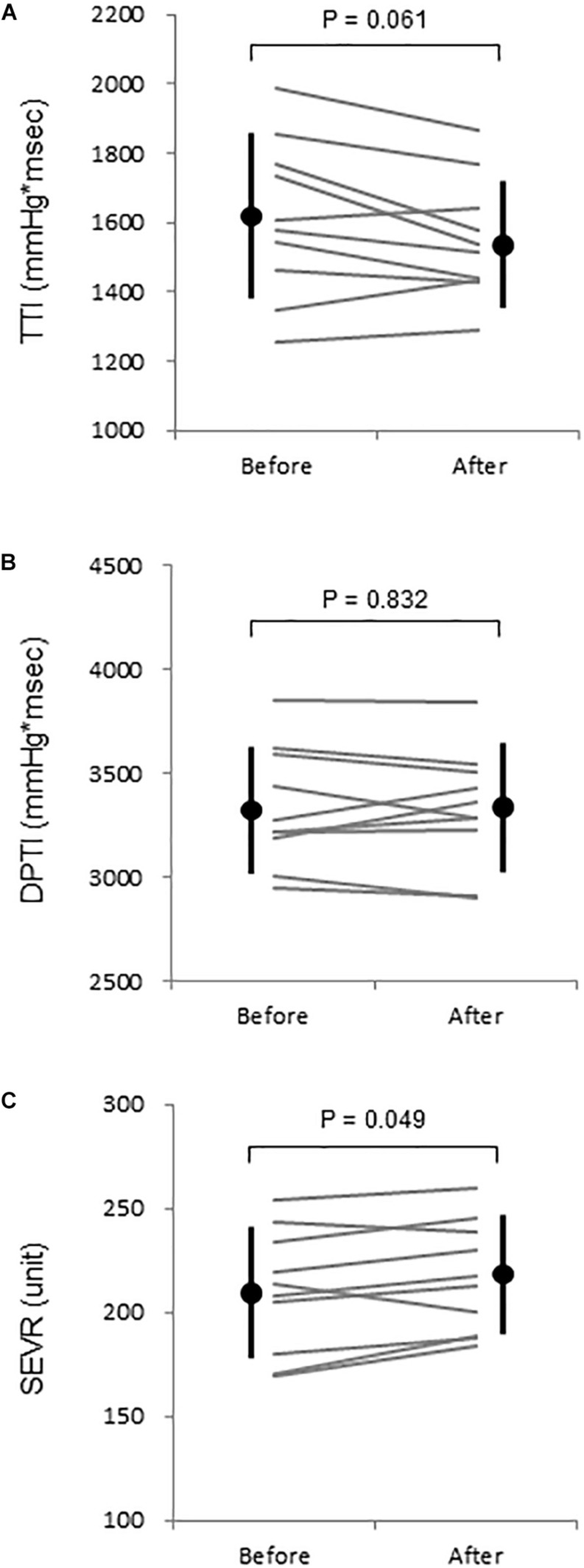
**(A)** Aortic tension-time index (TTI), **(B)** diastolic time-pressure index (DPTI), and **(C)** subendocardial viability ratio (SEVR) before and after warm water immersion. Circles and error bars are mean and SD. Gray lines indicate individual changes.

After the WWI, tympanic temperature did not change significantly (36.4 ± 0.4 vs. 36.4 ± 0.4°C, *P* = 0.879). Table 1 summarizes systemic and aortic hemodynamics before and after WWI. HR (*P* = 0.034) and brachial DBP (*P* = 0.043) slightly but significantly decreased, whereas there were no significant differences in SV (*P* = 0.694), CO (*P* = 0.431), TPR (*P* = 0.319), brachial SBP (*P* = 0.202), brachial PP (*P* = 0.900), aortic SBP (*P* = 0.565), aortic DBP (*P* = 0.300), aortic PP (*P* = 0.927), AP (*P* = 0.067), AIx (*P* = 0.167), and AIx_@HR__75__*bpm*_ (*P* = 0.398) between pre- and post-WWI. DPTI did not change significantly (*P* = 0.832), whereas TTI tended to be decreased (*P* = 0.061). Consecutively, SEVR significantly increased (*P* = 0.049, Power[1−β] = 0.72) (Figure 2).

**TABLE 1:** Systemic and central (aortic) hemodynamic variables before and after warm water immersion (WWI).

	Before WWI	After WWI	*P*-value
Heart rate, beats/min	55 ± 5	53 ± 6	0.034
Stroke volume, mL	95.4 ± 6.2	93.5 ± 15.7	0.694
Cardiac output, L/min	5.2 ± 0.6	5.0 ± 0.8	0.431
Total peripheral resistance	12.6 ± 2.4	13.5 ± 4.2	0.319
Brachial systolic BP, mmHg	110 ± 9	108 ± 9	0.202
Brachial diastolic BP, mmHg	69 ± 7	67 ± 6	0.043
Brachial pulse pressure, mmHg	41 ± 7	41 ± 7	0.900
Aortic systolic BP, mmHg	99 ± 8	98 ± 8	0.565
Aortic diastolic BP, mmHg	68 ± 7	68 ± 6	0.300
Aortic pulse pressure, mmHg	30 ± 5	30 ± 6	0.927
AP, mmHg	3.9 ± 2.8	4.8 ± 3.1	0.067
AP@HR_75__*bpm*_, mmHg	0.4 ± 2.7	1.1 ± 2.7	0.195
AIx,%	13.4 ± 10.0	15.7 ± 9.6	0.167
AIx@HR_75__*bpm*_,%	2.1 ± 8.6	3.6 ± 8.3	0.398

*Data are mean and SD. AP, augmentation pressure; AP@HR_75__*bpm*_, AP corrected by 75 beats/min of heart rate; AIx, augmentation index; AIx@HR_75__*bpm*_, AIx corrected by 75 beats/min of heart rate (HR); BP, blood pressure.*

**FIGURE 3:**
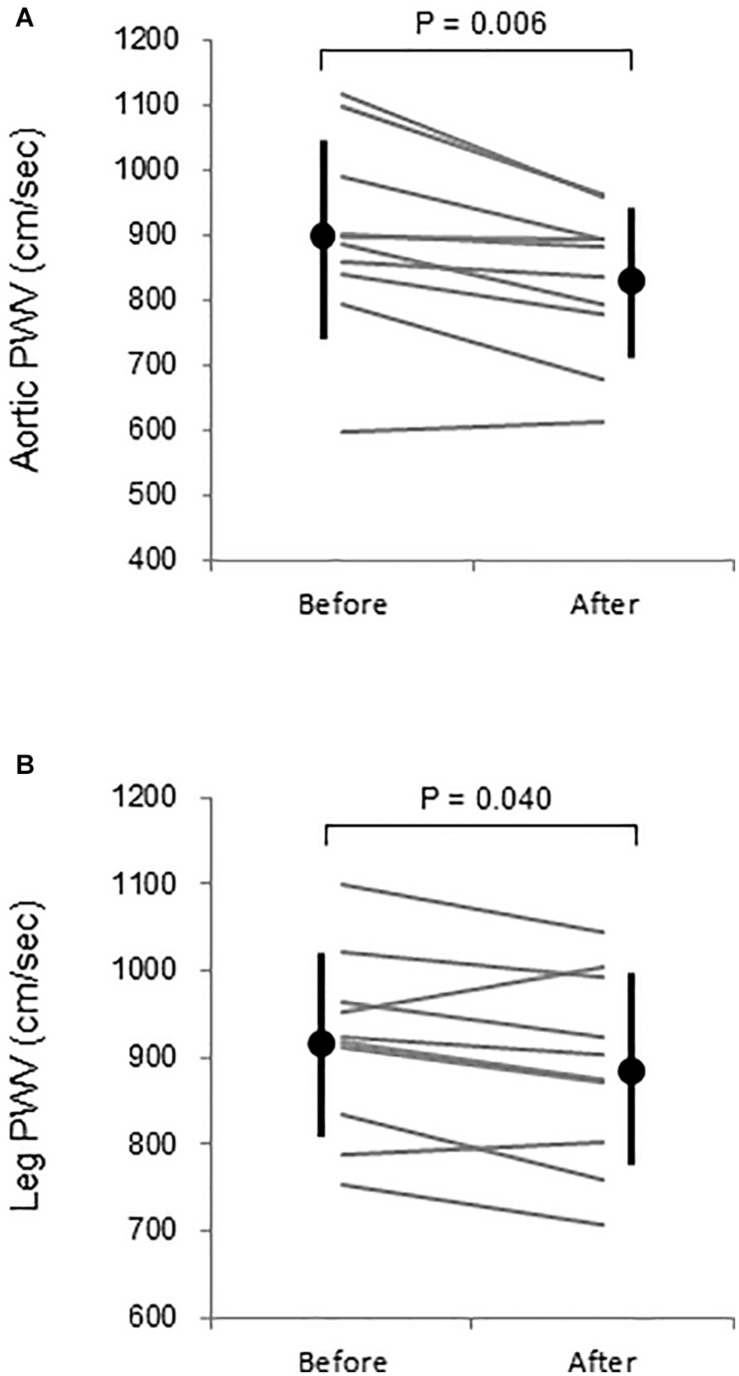
**(A)** Aortic and **(B)** leg pulse wave velocity (PWV) before and after warm water immersion. Circles and error bars are mean and SD. Gray lines indicate individual changes.

**FIGURE 4:**
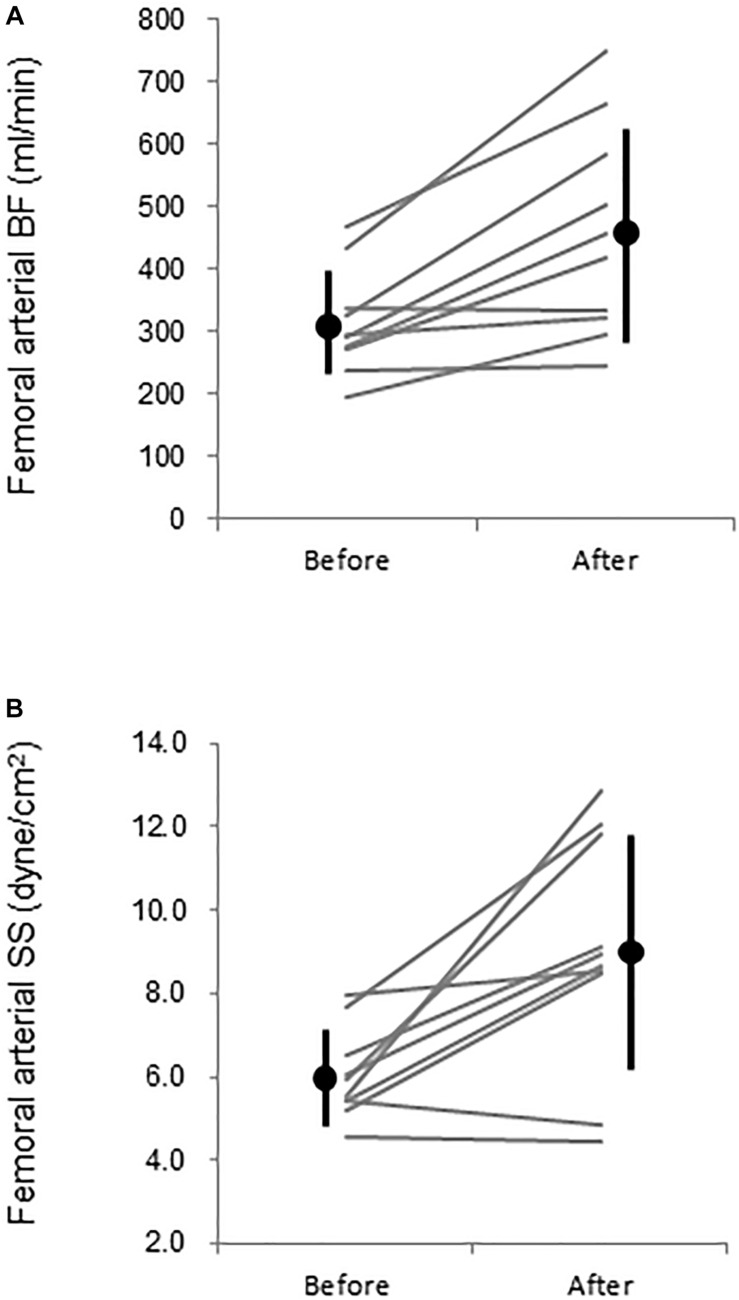
Femoral **(A)** arterial blood flow (BF) and **(B)** leg vascular resistance (VR) before (PRE) and after (POST) warm water immersion. Circles and error bars are mean and SD. Gray lines indicate individual changes.

**FIGURE 5:**
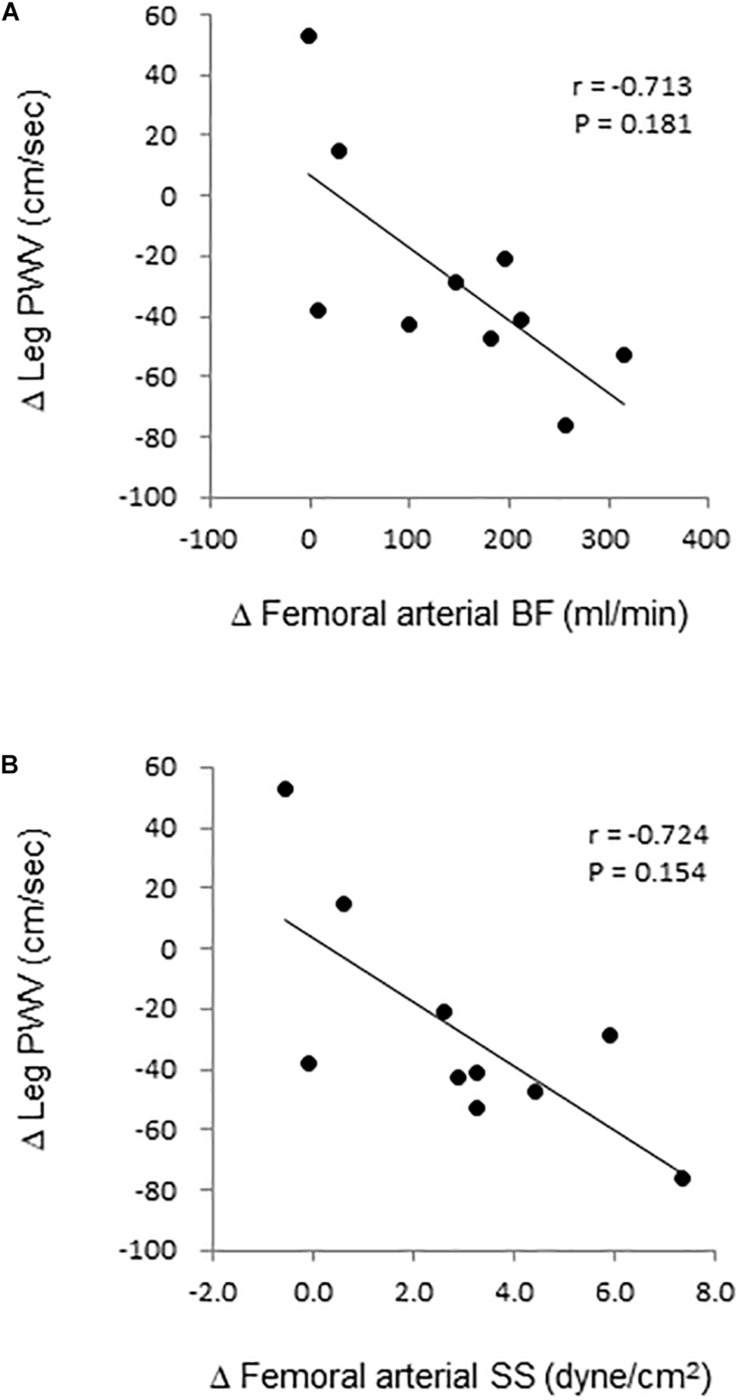
Relation. of change in leg pulse wave velocity (PWV) with corresponding changes in femoral arterial **(A)** blood flow (BF) and **(B)** shear stress (SS).

Aortic and leg PWV were significantly decreased by 7.5% (897 ± 150 vs. 830 ± 115 cm/s, *P* = 0.006, Power[1−β] = 0.88) and 3.1% (916 ± 104 vs. 888 ± 110 cm/s, *P* = 0.04, Power[1−β] = 0.57), respectively (Figure 3). Femoral arterial blood flow and SS were increased by 45.9% (*P* = 0.002, Power[1−β] = 0.78) and by 49.9% (*P* = 0.005, Power[1−β] = 0.928) (Figure 4). Change in leg PWV was significantly correlated with corresponding change in femoral arterial blood flow (*r* = −0.713, *P* = 0.181) and SS (*r* = −0.724, *P* = 0.154) (Figure 5).

## Discussion

The main results of this study are as follows. First, following 5-min WWI which did not elicit significant change in tympanic temperature, aortic and leg PWV were decreased; second, aortic BP and Alx did not change significantly but SEVR was increased. These results suggest that a short-term WWI acutely improves central and peripheral arterial stiffness and hemodynamics.

Large elastic arteries have important roles (called “Windkessel function”) to buffer the cyclic mechanical forces of cardiac pulsations, maintain coronary perfusion, and protect vulnerable microvasculature such as that in the brain (Nichols and McDonald, 2011). A reduction in this ability as manifested by increased arterial stiffness has been established as an independent determinant of cardiovascular and cerebrovascular risks (Laurent et al., 2003; O’Rourke and Safar, 2005; Vlachopoulos et al., 2010). To our knowledge, this is the first study that demonstrated that short-term (e.g., 5 min) WWI could acutely reduce central arterial stiffness in middle-aged men. Our results may partly support the previous finding of the chronic effect of WWI that repetitive bathing lowers central arterial stiffness (Brunt et al., 2016). Importantly, we empathize that SEVR was increased after WWI, suggesting improvement of coronary perfusion. Arterial destiffening is associated with an increase in Windkessel function. The expanded arterial wall seems to recoil well in diastole and prevent a substantial drop of diastolic pressure, a driving force of coronary perfusion.

We can speculate underlying mechanisms of decreased PWV. Arterial stiffness is determined by anatomical (e.g., elastin, collagen, calcium, and advanced-glycation end-product) and physiological (e.g., vasoactive substance, sympathetic nerve activity, BP, blood viscosity) components (Nichols and McDonald, 2011). Since a single bout of WWI is unlikely to acutely induce structural remodeling, the functional component would be attributed to the temporal reduction of arterial stiffness. As shown in Table 1, HR and CO were increased during 5-min WWI. The increased blood flow with WWI elicits a concomitant increase in SS (Naylor et al., 2011) and promotes endogenous production of vasodilators (e.g., nitric oxide) which are known to attenuate vascular tone and arterial stiffness (Kinlay et al., 2001; Bellien et al., 2010). Indeed, femoral arterial blood flow and SS remained substantially higher by 46% (vs. baseline before WWI) 10 min after WWI, and such change was significantly correlated with a corresponding change in leg PWV. These results imply that, at least in part, the increase in conduit artery SS is associated with WWI-induced reduction of arterial stiffness. Residual effects on blood flow and arterial stiffness have to be examined in a future study.

Several studies reported that heat stimulation which induced the elevation of core temperature did not reduce central arterial stiffness. It is well known that passive heat stress increases sympathetic activation (Low et al., 2011; Gagnon et al., 2015; Stojadinovic et al., 2015), and sympathetic activation is associated with increased arterial stiffness (Tanaka et al., 2017; Holwerda et al., 2019). Also, prolonged heat exposure elicits dehydration and elevation of blood viscosity which results in the increased PWV (Stojadinovic et al., 2015). These factors might mask the lowering effect of passive heat stimulation on arterial stiffness. On the other hand, we applied warm water (40–41°C) for only 5 min and could not observe a significant change in tympanic temperature. The improvement of central and leg arterial stiffness might be manifested by such an experimental design.

Experimental considerations should be mentioned. First, the present study confirmed a temporary change of arterial stiffness immediately after WWI (within 10–15 min). However, we did not observe its recovery (e.g., 20 min after the end of WWI). Besides, whether such change directly links to chronic adaptation is still unknown. Second, since PWV is influenced by BP, it might not reflect arterial stiffness appropriately when the concomitant BP change occurs (Benetos et al., 1993). However, in the present study, reduction of diastolic BP was small (−2 mmHg). In the case including the substantial BP change, BP-independent indices of arterial stiffness such as beta-stiffness index and cardio-ankle vascular index (CAVI) might be suitable (Hirai et al., 1989; Shirai et al., 2006). Third, since this study did not involve dry (no-water) and thermoneutral conditions, we could not clarify the impacts of water temperature and immersion separately. Also, we examined the effect of WWI at the manubrium of sternum level only based on the previous study (Brunt et al., 2016). To determine the effects of WWI at the other water levels (e.g., neck and lower body) would allow us practical important information. However, at least in part, our findings extend previous notion about potential of CVD prevention by WWI. Fourth, participants of this study had a daily bathing habit. Prior WWI experience may influence results (i.e., downregulation of alteration in vascular function including arterial stiffness and endothelial function). At last, a small number of healthy men were studied. To confirm the generalizability, future studies on other populations such as women, the elderly, and individuals with CVD risks, are required. In conclusion, we determined the acute effects of short-term WWI on arterial stiffness and central hemodynamics in healthy men. Our findings indicate that a short-term WWI acutely improves central and peripheral arterial stiffness and coronary perfusion. Further studies to determine the interaction between the residual effect of a single bout of WWI and chronic change (e.g., adaptation) in arterial stiffness and central hemodynamics are needed.

## Data Availability Statement

The raw data supporting the conclusions of this article will be made available by the authors, without undue reservation.

## Ethics Statement

The studies involving human participants were reviewed and approved by the institutional review board in National Institute of Advanced Industrial Science and Technology. The patients/participants provided their written informed consent to participate in this study.

## Author Contributions

JS decided on the conception and design of the research. JS and TT performed the experiments, analyzed the data, and interpreted the results of the experiments. JS prepared figures. JS drafted the manuscript. All authors edited and revised the manuscript, and read and approved the final manuscript.

## Conflict of Interest

The authors declare that the research was conducted in the absence of any commercial or financial relationships that could be construed as a potential conflict of interest.
