# Supplementary material for: Acute Effects of Short-Term Warm Water Immersion on Arterial Stiffness and Central Hemodynamics
Source: Front Physiol. 2021 Feb 4;12:620201. doi: 10.3389/fphys.2021.620201 (PMC7890244; doi:10.3389/fphys.2021.620201)
Supplement: Supplementary file 1 [file Table_1.pdf]

Supplemental Table 1. List of hemodynamic measurements

Systemic hemodynamics

Heart rate (HR, via electrocardiogram)

Brachial blood pressure (via a non-invasive blood pressure measurement device)

Stroke volume (SV, via Modelflow method)

Cardiac output (CO = SV × HR)

Total peripheral resistance (TPR = mean arterial pressure / CO)

Arterial stiffness

Aortic pulse wave velocity (aortic PWV, via a vascular testing device)

Leg pulse wave velocity (leg PWV, via a vascular testing device)

Central hemodynamic variables.

Carotid blood pressure (via applanation tonometry)

Aortic blood pressure (via the generalized transfer function)

Aortic augmentation pressure (AP, via pulse wave analysis)

Aortic augmentation index (AIx, = AP / aortic pulse pressure × 100)

Time-tension index (TTI, via pulse wave analysis)

Diastolic pressure-time index (DPTI, via pulse wave analysis)

Subendocardial viability ratio (SEVR = DPTI / TTI × 100)

Leg hemodynamics.

Femoral artery blood flow velocity (BFV, via an ultrasound machine)

Femoral artery blood flow (= mean BFV × [circular area] × 6×10^4)

Femoral arterial shear stress (SS, = 8 × blood viscosity × mean BFV / diameter)
